# Biological Control of Mosquito-Borne Diseases: The Potential of* Wolbachia*-Based Interventions in an IVM Framework

**DOI:** 10.1155/2018/1470459

**Published:** 2018-11-15

**Authors:** El Hadji Amadou Niang, Hubert Bassene, Florence Fenollar, Oleg Mediannikov

**Affiliations:** ^1^VITROME, Campus International UCAD-IRD, Dakar, Senegal; ^2^Aix-Marseille Univ, IRD, AP-HM, MEPHI, IHU-Méditerranée Infection, Marseille, France; ^3^Laboratoire d'Ecologie Vectorielle et Parasitaire, Faculté des Sciences et Techniques, Université Cheikh Anta Diop (UCAD) de Dakar, Senegal; ^4^Aix Marseille Univ, IRD, AP-HM, SSA, VITROME, IHU-Méditerranée Infection, Marseille, France

## Abstract

People living in the tropical and subtropical regions of the world face an enormous health burden due to mosquito-borne diseases such as malaria, dengue fever, and filariasis. Historically and today, targeting mosquito vectors with, primarily, insecticide-based control strategies have been a key control strategy against major mosquito-borne diseases. However, the success to date of such approaches is under threat from multiple insecticide resistance mechanisms while vector control (VC) options are still limited. The situation therefore requires the development of innovative control measures against major mosquito-borne diseases. Transinfecting mosquitos with symbiotic bacteria that can compete with targeted pathogens or manipulate host biology to reduce their vectorial capacity are a promising and innovative biological control approach. In this review, we discuss the current state of knowledge about the association between mosquitoes and* Wolbachia*, emphasizing the limitations of different mosquito control strategies and the use of mosquitoes' commensal microbiota as innovative approaches to control mosquito-borne diseases.

## 1. Introduction

Mosquitoes of the* Anopheles*,* Aedes*, and* Culex* genera include a number of main vector species of protozoan, virus, and nematode pathogens [1]. Therefore, since their first association with the transmission of such pathogens to humans and other vertebrates in the late nineteenth century [1], targeting mosquito vectors to interrupt the transmission of diseases has been a key control strategy against major mosquito-borne diseases such as malaria, yellow fever, dengue, chikungunya fever, and Zika virus infection. During the first quarter of the twentieth century, mosquito control activities were primarily based on source reduction, through larviciding using petroleum oils and larvivorous fish, together with environmental-based management [2]. With the advent of Dichlorodiphenyltrichloroethane (DDT) and the discovery of its insecticidal properties in the early 1940s, began the chemical era of vector control (VC) with mainly DDT-based interventions, both as larvicide and adulticide [3]. The publication of “*Silent Spring*” by Rachel Carson in 1962 raised public concerns about the use of DDT, characterized by high mammalian toxicity, poisoning risks to nontarget organisms, persistence in the biosphere surface, and an accumulation in food-chains [2]. Increasing public concerns about Persistent Organic Pollutants (POPs) led to DDT being banned. Fortunately, in the 1980s, a few years before the prohibition of DDT, synthetic pyrethroids compounds were added to the arsenal of public health insecticides [4]. Between 2000 and 2015, pyrethroid-treated bed-nets (ITNs), indoor residual spraying (IRS) with residual insecticides, and other insecticide-based strategies were widely used as front-line tools against the vectors of malaria and other mosquito-borne diseases [5] in an Integrated Vector Management (IVM) framework [3]. However, despite controversial, growing multiple insecticide resistance mechanisms threaten to reverse the progresses made so far to eliminate or control main mosquito-borne diseases [1, 6]. In this context, attention has turned toward research onto biological control, transgenic and paratransgenic approaches as potential alternatives, or complements to current chemical strategies [7].

Mosquito transgenesis is based on genetic modifications to introduce novel elements into the mosquito genomes. According to Abraham et al. [8], the two major transgenic approaches are (i) the genetic suppression or limitation of the vectors' ability to serve as competent hosts for parasite development, thus decreasing or eliminating their ability to transmit pathogens (vector competence), and (ii) the genetic suppression of insect populations by reducing the lifespans of known vectors. These approaches can, potentially, be used to control mosquito populations by reducing their ability to transmit human or animal pathogens [9]. For instance,* Anopheles gambiae* and* Anopheles stephensi*, the respective main malarial vectors in Africa and Asia, have been successfully engineered to interfere with malaria parasites, to stop or at least reduce transmission of the disease [10, 11]. Nowadays, there is a huge potential for transgenic vector control strategies. However, genetic manipulation tends to reduce the fitness of the modified mosquitoes thus reducing the chance of successfully spreading of genes of interest among natural populations of the targeted vector species [12]. Moreover, failure of spreading transgenes (Weill M. personal communication), mutation, and recombination rates could seriously undermine the feasibility or durability of such an approach as anticipated for the engineered geminivirus aiming to protect crops in the agricultural sector [5].

The recent discovery of a number of symbiotic bacteria inhabiting the gut and/or reproductive tissues of arthropods has opened the way for innovative control strategies against some of the major vector-borne diseases [13, 14]. Indeed, bacterial symbionts associated with mosquitoes can directly exert a pathogenic effect on their host [15], interfere with its reproduction [16, 17], and reduce vector competence [18]. Furthermore, the use of genetically modified bacteria to deliver antiparasite molecules has several advantages over the use of genetically modified vectors [19]. Strategies to exploit symbiotic microorganisms to control vector-borne diseases are known as paratransgenesis, i.e., the generation of engineered symbionts expressing antiparasite molecules [20]. Moreover, organisms that are able to manipulate their host biology and even shorten their lifespan may be of the highest interest for use as biological control agents.

Over the last decade, the focus has been put upon symbiotic microorganisms to identify potential candidates which could be used in new vector control approaches. Among the most promising candidates, several strains of the genus* Wolbachia,* a dominant endosymbiotic bacterium of numerous insects including major vectors of zoonotic pathogens, are of highest interest for the scientific community. Indeed,* Wolbachia* is a maternally inherited that can infect mosquitoes' reproductive organs to self-sustain itself in host populations, but also somatic tissues where pathogens development occurs and compete with them. It is, therefore, an interesting biological control agent which can be used to stop or prevent the transmission of several vertebrate pathogens to humans and domestic animals [21].

In this review, we discuss the current state of knowledge about the association between mosquitoes and* Wolbachia*, emphasizing the limitation of different mosquito control strategies and the use of mosquitoes' commensal microbiota as innovative approaches to control mosquito-borne diseases.

## 2. Methodology

### 2.1. Search Methods

Peer-reviewed literature search was conducted using online databases including PubMed, Bibliovie, INSERM databases, Web of Knowledge, and Google Scholar for articles. Gray literature searches were conducted using World Health Organization (WHO) webpage. The key search terms used was different combinations of “mosquito”, “wolbachia”, “biological control”, “control”, and “mosquito-borne diseases” using the Boolean operator “OR”, and combinations between concepts used the logical “AND”.

### 2.2. Data Screening

All documents were quickly checked to assess their relevance to the project using information in the title and the abstract. A subset of all relevant documents was selected, sorted by section, further reviewed and compiled in the manuscript.

## 3. Main Text

### 3.1. The Genus of Wolbachia (Alphaproteobacteria)

#### 3.1.1. Description, Classification and Phylogeny

Bacteria of the genus* Wolbachia* are obligate intracellular Gram-negative bacteria belonging to the* Alphaproteobacteria* class (Table 1) found into the cytoplasmic vacuoles inside the cells of their insects, isopods, mites, arachnids and nematodes hosts [22]. The genus was first discovered in 1924 by Marshall Hertig and S. Burt Wolbach in the reproductive organs of* Culex pipiens* [23], then subsequently described by Hertig in 1936, who named the genus after his collaborator [24].

*Wolbachia pipientis* is the unique valid species of the genus. Noteworthily, the two other species that have been previously described as belonging to the genus* Wolbachia* [25]:* Wolbachia melophagi* and* Wolbachia persica* were removed latter on [26].* W. melophagi* is now considered as* nomen nudum*, because no strain of this species has been found to date. While* W. persica*, which was isolated from the soft tick* Argas persicus*, was erroneously attributed to the genus as revealed by phylogenetic analysis of the 16S rRNA gene showing its close relatedness to the genus* Francisella* [27].

**Figure 1:**
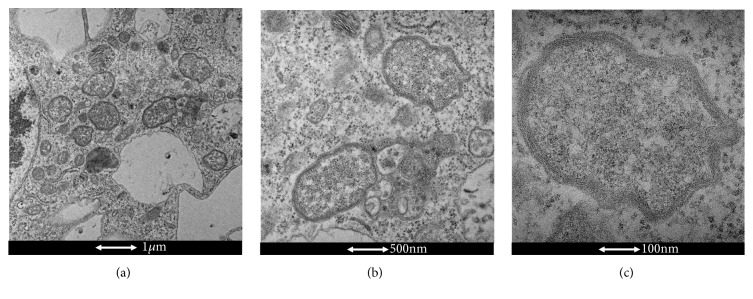
**Electron microscopy of* Wolbachia*. **(**a**)* Wolbachia* cocci (Scale bar: 1 *μ*m). (**b**) Zoom of two* Wolbachia* cells (Scale bar: 500 nm). (**c**) Zoom of a single* Wolbachia* cell (Scale bar: 100 nm). (by El Hadji Amadou Niang).

Morphologically,* Wolbachia* is a pleomorphic bacterium (Figure 1) that appears as small rods [0.5–1.3 *μ*l in length] and coccoid forms [0.25–1 *μ*l]; large forms [1–1.8 *μ*l in diameter] growing inside vacuoles in the cytoplasm of host cells [28]. Despite its Gram-negative cell wall structure,* Wolbachia *is poorly stained by Gram staining, but can be well visualized using the Diff-Quik and May-Grunwald-Giemsa staining methods. Using the Gimenez stain, they can also be visualized as dark-blue structures within a blue-green cytoplasm [29]. Since they do not form* morulae* and exclusively infect arthropods and filarial nematodes,* Wolbachia* are easily differentiated from other closely related genera [28].

**Figure 2:**
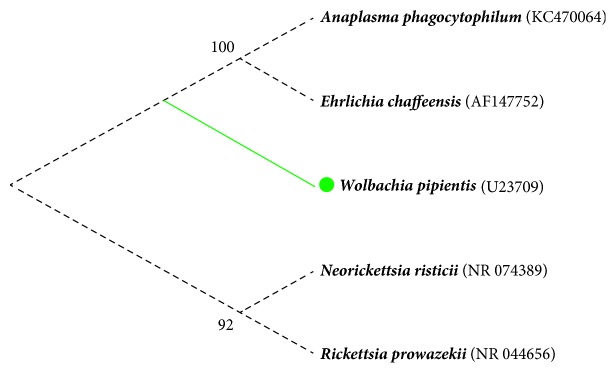
**Molecular Phylogenetic analysis of* Anaplasmataceae* by Maximum Likelihood method.** The evolutionary history was inferred using the Maximum Likelihood method based on the Tamura-Nei model [30]. The tree with the highest log likelihood (-4338.5700) is shown. The percentage of trees in which the associated taxa clustered together is shown next to the branches. Initial tree(s) for the heuristic search were obtained automatically by applying the Neighbor-Join and BioNJ algorithms to a matrix of pairwise distances estimated using the Maximum Composite Likelihood (MCL) approach, and then selecting the topology with the higher log likelihood value. The analysis involved five nucleotide sequences. All positions containing gaps and missing data were eliminated. There was a total of 1,411 positions in the final dataset. Evolutionary analyses were conducted in MEGA7 [31].

Phylogenetic analysis of the 16S rRNA gene (Figure 2), showed that* W. pipientis*, the* nomen* of the genus, forms a monophyletic clade within the* Alphaproteobacteria* class, closely related to the* Anaplasma*,* Ehrlichia* and* Neorickettsia* genera of the* Anaplasmataceae* family [28].

Further analysis based on the 16S rRNA and the* Wolbachia* Surface Protein (*wsp*) genes, were used to cluster the species into finer taxonomic scales. A system based on the level of similarity in the* wsp* gene sequences has been proposed for strain grouping. So far, 16 main evolutionary lineages from different host taxa known as “supergroups” have been identified and designated by the letters A to Q, with the exception of G [22]. The usefulness of such an assemblage remains controversial and the suggestion of splitting* W. pipientis* into multiple species has its pros and cons [32, 33].

Typically, the supergroups A and B are widely spread across many arthropod taxa [34]. Their common ancestor has probably diverged approximately 58–67 million years ago, at a time when all modern* Arthropoda* orders already existed [22]. The supergroups C and D are obligate and beneficial endosymbionts in some filarial nematodes [34–37]. While the F supergroup is peculiar and includes both nematode and arthropod* Wolbachia* strains [37–40]. More specific to certain host lineages, strains in supergroup E have been reported from* Collembola* [41, 42], in H from termites [40], and in M and N from aphids [43]. Further distinct supergroups have been identified either in nematodes or arthropods [34].

#### 3.1.2. Obligate Intracellular Lifestyle

A range of microbial pathogens interact with their host in numerous and complex ways. Many are extracellular, while others invade organs and multiply within specific vector cells [44].* Wolbachia* belongs to the latter group and has an original lifestyle as an obligate intracellular symbiont (endosymbiont) in close relationship with infected eukaryotic cells [45]. In arthropods,* Wolbachia* grow inside vacuoles often within the cytoplasm in the host's reproductive cells. However, they can also be found in somatic tissues, including nervous tissue and hemocytes [28]. Growing research has provided exciting insights into various aspects of the* Wolbachia*'s biology [46]. One of the most obvious consequences of their presence inside reproductive cells is the facilitation of their transovarian transmission to their host's offspring. Analysis of the sequenced genomes of several members of the* a-Proteobacteria *group, to which belong the* Wolbachia* genus, has also provided greater understanding of their reductive genome evolution and antigenic variation as well as how they manipulate host cells [44]. However, the intracellular lifestyle has led to the loss of several genes as a consequence of the reduced genome size, varying from 1.1 Mb to 1.5 Mb, including less than 1000 protein-coding genes [45]. Furthermore, it has been reported that intracellular symbionts, such as* Wolbachia*, transfer genes into the host nucleus and vice versa [47, 48]. Leclercq et al. [49] showed high affinity between coding sequences of the* f-element* of the common pillbug (*Armadillidium vulgare*) with a large piece of the genome of the feminizing wVulC* Wolbachia* strain. Symbionts may also acquire genes from other symbionts [47]. The high level of genetic exchange in* Wolbachia* mentioned above suggests that its core genome is completed by an extensive auxiliary genome. As explained by Ishmael et al. [50], the core genome contains all the housekeeping genes shared by all (or almost all) sequenced strains for a given taxon, while all other genes constitute the auxiliary genome, encompassing the genetic variation within the species.

#### 3.1.3. Host Reproductive Manipulation

**Figure 3:**
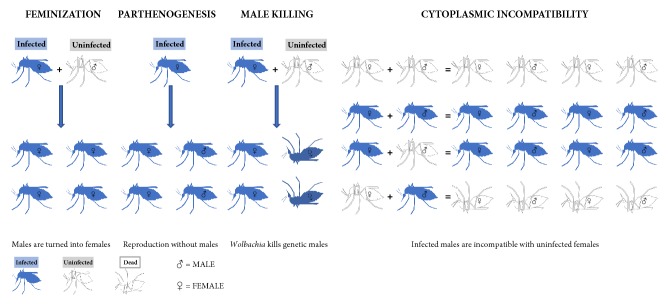
Different phenotypes of* Wolbachia*'s host reproductive manipulation.

*Wolbachia *are typically transmitted vertically through host eggs and alter host biology in diverse ways. They induce reproductive manipulations (Figure 3), such as (i) the feminization of infected males (i.e., turning genetic males into females); (ii) induced parthenogenesis (i.e., reproduction without males); (iii) killing of infected males; and (iv) cytoplasmic incompatibility (i.e., modification of sperm from infected males resulting in embryonic defects and death) [34, 51].

*(1) Feminization*. Infected males are “dead ends” for* Wolbachia* inheritance, because they do not transmit* Wolbachia* infection to their offspring. Thus, converting infected male offspring into females increases the potential for* Wolbachia* to be transmitted to the next generation. The phenomenon was first described in isopods such as* Armadillidium vulgare* and more recently in insects [52], where it involves different mechanisms, operating at the embryonic stage [51, 52]. In several species of terrestrial isopod within the order* Oniscidae*,* Wolbachia* invade the androgenic gland. The hypertrophied gland is then inhibited, causing genetic males to develop as females [53]. Among insects, feminizing strains have been reported in* Ostrinia furnacalis* (*Lepidoptera*) and in* Eurema hecabe* (*Lepidoptera*) and* Zyginidia pullula* (*Hemiptera*), in which the involved mechanisms remain unclear [51].

*(2) Parthenogenesis*. Another beneficial strategy to increase the maternal inheritance of* Wolbachia* is to induce the production of female offspring without fertilization by sperm, a process known as parthenogenesis (thelytoky).* Wolbachia*-induced female parthenogenesis is less common and has only been documented in haplodiploid species such as thrips (*Thysanoptera*), mites (*Acari*) and wasps (*Hymenoptera*) [51]. In these organisms, males normally develop from unfertilized haploid eggs (arrhenotokous parthenogenesis), whereas females develop from fertilized diploid eggs.* Wolbachia* disrupt the cells' early embryonic development, doubling the number of chromosomes in the unfertilized haploid eggs and rendering them diploid. This leads to development as an asexually produced female, so that infected females produce twice as many daughters as uninfected ones, allowing their cytoplasm to be transmitted to twice as many granddaughters as possible [54].

*(3) Male Killing*. In* Coleoptera*,* Lepidoptera*,* Diptera* (*Insecta*) and* Pseudoscorpiones* (*Arachnida*),* Wolbachia* induce male killing of infected females' male progeny. This phenotype, occurring mainly during embryogenesis, provides fitness benefits to the female progeny in terms of the competition for resources. -induced male killing occurs through lethal feminization. Indeed, when Insight into the mechanism has shown that* Wolbachia* infected mothers were treated with tetracycline to remove* Wolbachia*, genetic males survive, whereas in the presence of* Wolbachia*, genetic males become feminized and die during larval development [51, 54].

*(4) Cytoplasmic Incompatibility (CI)*.* Wolbachia*-induced cytoplasmic incompatibility (CI) is the most commonly described reproductive manipulation phenotype. Reproductive incompatibility between populations of the* Culex pipiens* mosquito was first reported in the 1950s, but* Wolbachia* was only identified as the causative agent in the 1970s [55]. This phenotype comprises two distinct components:* Wolbachia*-induced modification of sperm during spermatogenesis and rescue of this modification in embryos infected with the same strain [51]. The incompatible cross, due to the asynchrony of the male and female pronuclei phases at the initial stage of mitosis, occurs when* Wolbachia*-infected males mate with uninfected females (unidirectional CI). Bidirectional CI occurs when both partners are infected by different but incompatible* Wolbachia* strains, causing cross lethality in both directions. CI has been widely described in numerous arthropod host species infected by* Wolbachia* strains belonging to both the A and B supergroups [56].

#### 3.1.4. *Wolbachia *spp. and Insects

**Figure 4:**
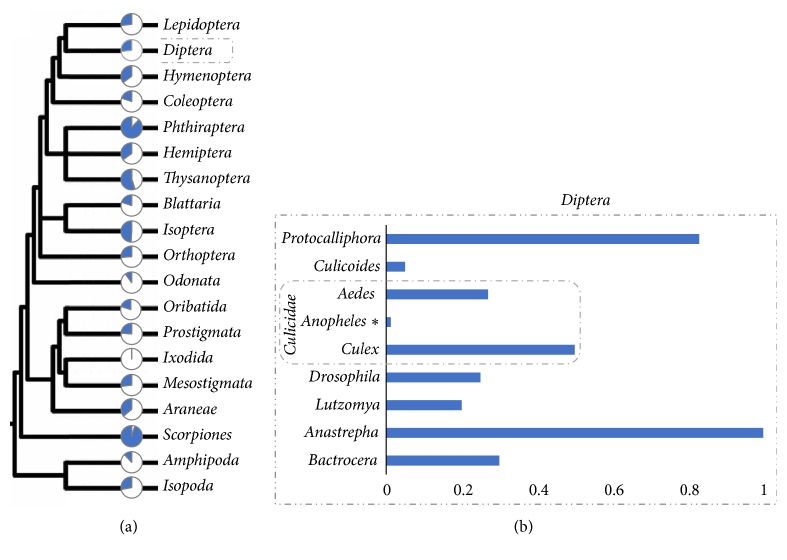
***Wolbachia* in arthropods**. (Modified from Russell & Steiner 2012, Myrmecological News Journal [57]). (a) Graph illustrating* Wolbachia*-infected (Blue shaded portion) and* Wolbachia*-uninfected (white portion) proportions by host taxon. (b) Histogram highlighting the frequencies* Wolbachia* infection of some dipteral families. The asterisk (*∗*) indicates the recent discovery of native* Wolbachia* within the* Anopheles* genus.

The “pandemic” nature of* Wolbachia* [46] resulting in their widespread distribution in various invertebrate hosts (Figure 4) is explained by their ability to manipulate host reproduction, but also by their ability to move horizontally across species boundaries [51, 54]. It has been estimated that different strains of* Wolbachia* may infect more than 65% of insect species [34]. Among these, several mosquito species belonging to different genera have been found carrying different strains.

### 3.2. Mosquitoes (*Diptera*,* Culicidae*)

#### 3.2.1. Taxonomy, Classification and Phylogeny

**Figure 5:**
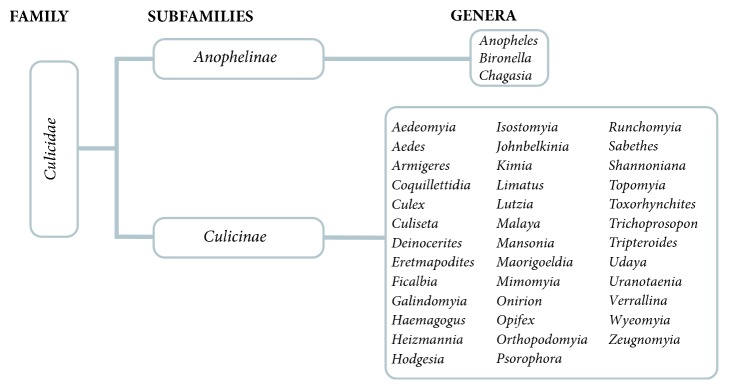
**Classification of mosquitoes (DIPTERA: CULICIDAE)** (by El Hadji Amadou Niang).

Mosquitoes are a monophyletic group that belongs to the order of* Diptera* (Table 2 and Figure 5) [58]. The origin and phylogenetic history of the family of* Culicidae* dates back to the Mesozoic Era. It is estimated that the main lineages of current mosquitoes date from the early Cretaceous period (145-100 million years) [58, 59].

The family of* Culicidae* is a large and abundant group which is distributed from tropical latitudes to temperate regions, well beyond the Arctic Circle. It includes approximately 4,000 species, classified into two subfamilies and 112 genera. The subfamily* Anophelinae* has three genera and* Culicinae* has 109 genera, segregated into 11 tribes [58].

Mosquitoes are of prime medical and veterinary importance. In nearly all* Culicidae* species, only females feed on vertebrates, because of their need for blood to produce their offspring. During blood-sucking, a complex salivary secretion facilitates feeding but also enables several pathogens (viruses, protozoa, and nematode worms) to be directly injected into the capillaries of their vertebrate hosts [60].

#### 3.2.2. Mosquitoes of Medical and Veterinary Importance

Mosquito-borne diseases such as malaria, filariases, dengue, chikungunya, Zika, and West Nile fevers represent significant medical and veterinary problems around the world and lead to major economic problems [61].  Table 3 summarises some of the most devastating mosquito-borne diseases [59].

#### 3.2.3. *Wolbachia* and Mosquitoes

Among* Culicidae*, two types of* Wolbachia* infections can be distinguished: natural* Wolbachia* infections and transinfected mosquito lines.

*(1) Natural Wolbachia Infections*. The interest in the* Wolbachia* genus has renewed when the biological connection between cytoplasmic incompatibility and* Wolbachia* infection was established and documented by Yen & Barr [55] in the early 1970s. Subsequently, Yen [62] reported the presence of* Wolbachia* within the ovaries and eggs of mosquito members of the* Aedes scutellaris* group (*Aedes cooki*,* Aedes polynesiensis*,* Aedes albopictus*, and* Aedes riversi*). In 2002, while screening several mosquitoes species, Ricci et al. [63] found arthropod strains of* Wolbachia* in* Culex modestus*,* Culex pipiens, *and* Coquillettidia richiardii*, while three other mosquitoes (*Aedes cinereus*,* Aedes detritus, *and* Ae. geniculatus*) were infected with filarial strains previously described from* Dirofilaria immitis*, and two mosquitoes (*Aedes punctor *and* Culex torrentium*) were positive for both arthropod and filarial strains. Later, the development of PCR and sequencing techniques has led to the discovery of many other* Wolbachia* strains from several other mosquito species.

A recent meta-analysis of the distribution of* Wolbachia* in mosquitoes indicated that, of 185 mosquito screened, 31.4% was* Wolbachia*-infected but also demonstrated the nonrandom distribution of* Wolbachia* among different mosquito taxa [64]. Indeed,* Wolbachia* was found in 39.5% of the 147 mosquito species screened, but never in* Ae. aegypti*, the primary vector of Dengue, Chikungunya, and Zika viruses [9, 64]. Moreover, prior to 2014 no* Wolbachia* infection has been documented in 38 anopheline species, including several important malarial vector species (*An. gambiae*,* An. arabiensis*,* An. funestus*,* An. stephensi*,* An. culicifacies*,* An. dirus*,* An. Albimanus,* and* An. darlingi*), which has led to the previous believe that* Wolbachia* were unable to infect* Anopheles* species until their very recent discovery in natural populations of* Anopheles gambiae* and* Anopheles coluzzii* in Burkina Faso [21, 65] and in Mali (Gomes et al. 2017). More recently Ayala et al. [66] and Jeffries et al. [67] have revealed that native* Wolbachia* infections was wider than expected among natural anopheline populations with at least 16 species naturally infected (Table 4). Furthermore, Ayala et al. [66] revealed a large diversity of* Wolbachia* strains in wild anopheline populations, which offers an unexpected opportunity to discover suitable phenotypes to suppress* Plasmodium* transmission and/or to manipulate* Anopheles* reproduction and reduces the malaria burden in Africa [66].

*(2) Mosquito Transinfection*. The absence of natural infection in some dominant vector species has been a limiting factor for the potential operational use of* Wolbachia* to control vectors and the diseases they transmit. The ability of the bacterium to adapt to new intracellular environments has been exploited to transinfect vector species of medical and veterinary importance [9]. Transinfection via embryonic microinjection was used to transfer several* Wolbachia* strains into* Ae. albopictus* [64] and* Ae. aegypti*. For instance, the life-shortening strain of* Wolbachia* (wMelPop-CLA) from* Drosophila melanogaster* was successfully and stably introduced into* Ae. aegypti *to reduce its life-span. Given the proof that wMelPop strain being protective against RNA viruses in Drosophila, its derivate has been used latter to block Dengue, Chikungunya transmitted by* Ae. aegypti*, while the wMel* Wolbachia* strain (wMel_Br) has been used successfully against Zika infections in Brazil [69, 70]. Contrary to the complex* Wolbachia*-Arbovirus vectors, current views about the impact of* Wolbachia* on* Plasmodium* infections are almost entirely based on artificially transfected mosquito models [71]. In the* Anophelinae* sub-family,* Wolbachia* transinfection has been successful in* Anopheles gambiae* [72, 73] and in* Anopheles stephensi* [74], respectively major vectors of human malaria in Africa and the Middle East, and South Asia [75, 76].

#### 3.2.4. Vector Control Approaches for the Control of Mosquito-Borne Diseases

In the past century, significant advances have been made in controlling main vector-borne diseases. Malaria disappeared from the northern hemisphere, diseases such as typhus, Bartonella, and yellow fever prevalence were drastically reduced in many countries with effective vector control methods [9]. Despite these successes, there are currently no effective vaccines against dengue, filariasis, or malaria, and specific treatments are only available for malaria and some filariases. Historically and today, targeting mosquito vectors has been a key control strategy against major mosquito-borne diseases. Vector control is an essential component of mosquito-borne disease prevention and control. Its aim is to interrupt or eliminate local transmission or reduce vulnerability to disease and prevent secondary infections from introduced diseases and prevent outbreaks. Before the Second World War, vector control was predominantly based on the environmental control of the proliferation of mosquitoes [3]. The so-called “*chemical period*” then began with the advent of DDT and other organochlorine pesticides in the late 1940s. During this period, widescale spraying of the indoor surfaces of houses and shelters drastically reduced the number of malarial mosquitoes and other insects and led to the successful eradication of malaria in the United States, European countries, the Soviet Union, South East Asia, India, and South America [4, 77]. But the Malaria Eradication Programme failed in several malarial pilot areas in the African continent, due to the extremely high malaria heterogeneity and vector behavioral plasticity [78]. However, during the past decade there has been a global renewed focus on vector control with the widespread use of impregnated (LLINs) and sprayed materials (IRS), particularly against malaria vectors. Large community-based distribution and/or IRS campaigns have led to significant ITN and IRS coverage in several African countries, resulting in a substantial drop in the prevalence of malaria in that region [3]. However, to make vector control more effective, cost effective, ecologically sound, and sustainable the WHO adopted in 2004 the Global Strategic Framework on Integrated Vector Management (IVM) as the first step towards the search and the implementation of new approaches to control vectors and the diseases they transmit [3]. Defined as “a rational decision-making process for the optimal use of resources for vector control,” IVM is not a new concept since its basic principles have been used over the past century in the USA through the vast network of Mosquito Abatement Districts implemented to protect people from nuisance-biting and vector species of mosquitoes [79]. Lately, the WHO called for the strengthening of IVM as one of the strategic areas for action in the global plan framework to combat neglected tropical diseases for 2008–2015.

Although insecticides have been successful in controlling vectors, current ecological and environmental protection standards make insecticide-based strategies unsustainable, due to the adverse effects of many insecticides on nontarget species, their environmental impact, the contamination of soil and water and the development of selective processes, and subsequent mosquito resistance to insecticides [1]. Moreover, a number of malaria prevention and control tools currently available are quite expensive, while arbovirus vector management also has to face significant challenges, due to the peculiar traits of* Aedes* vectors, which have huge physiological and ecological plasticity making them difficult to control [80]. A broad spectrum of resistance to insecticides has evolved in the* Culex* genus, involving both “Metabolic” (enhanced esterase, glutathione-S-transferase, or p450 monooxygenase activities) and “Target Site” (modification of the acetylcholinesterase; the GABA receptors; or the sodium channels) mechanisms [81]. There is, therefore, an urgent need for effective alternative vector control strategies that can be used on a large scale and which are environmentally friendly. This is critical to sustaining control efforts and to achieving the goal of malaria elimination. Potential alternatives or complementary strategies to current core interventions include genetic control approaches, using refractory mosquitoes to replace vector populations or the release of mosquitoes carrying a lethal gene to suppress the targeted populations [1]. In addition to transgenic mosquitoes, paratransgenic and biological control approaches provide concrete possibilities for innovative vector control strategies [7].

#### 3.2.5. Biological Control of Mosquito-Borne Diseases

Beyond the paratransgenic VC approaches taking advantage of the naturally/transinfected mosquitoes microbiota and defined as the use of symbiotic organisms naturally inhabit or successfully introduced into mosquitoes to deliver an effector molecule to inhibit, compete or kill the pathogen in insects [1, 9], their utilisation to directly interfere with or modulate vector immunity against pathogens constitutes a Biological approach to control MBD. The feasibility of the paratransgenic approach was demonstrated by Durvasula et al. [82], when they successfully transformed a commensal symbiont in the hindgut lumen of* Rhodnius prolixus*,* Rhodococcus rhodnii*, to express the cecropin A protein to kill the causative agent of Chagas disease, and* Trypanosoma cruzi* inside their host. Similarly, the recent use of the life-shortening* Wolbachia* wMelPop-CLA strain is a prelude for an innovative Biological approach to control MBD. Indeed, intracellular bacteria such as* Wolbachia* that can manipulate their host biology, including their immune system, are unduly regarded as promising innovative biocontrol approach to control insect-transmitted diseases. Therefore, several studies have attempted to show the potential for* Wolbachia* to be used in such a strategy to control mosquito-transmitted diseases [74].* Wolbachia* has several characteristics, including the capacity to perturb insect ecology, behaviour, and physiology, making it one of the best candidates for blocking, or at least significantly reducing, the transmission of pathogens of medical and veterinary importance [21]. However, before the operational implementation of any* Wolbachia*-based approach, an important prerequisite is to better characterize all potential the strain of the genus and their host manipulation phenotypes which could make them good biocontrol agents candidates, to develop predictive models, and to perform a comprehensive risk assessment for their use to control mosquitoes and disease they transmit. As stated before,* Wolbachia*-transinfection technology has already shown promise in controlling the transmission of arboviruses by* Ae. aegypti* using different* Wolbachia* strains which can shorten vector lifespans, limit susceptibility to infection, and induce cytoplasmic incompatibility to reduce vector density. Furthermore, in* An. gambiae* and* An. stephensi*, the presence of* Wolbachia* appears to negatively impact the* Plasmodium* developmental cycle and egg laying [21, 74, 83]. Although potentially eligible as an inovative weapon, our knowledge of* Wolbachia*-mediated antiparasite mechanisms is fragmented, if not completely lacking. A significant delay in the virus-induced mortality of the pathogenic Drosophila C, Cricket paralysis and Flock House virus have been related to the presence of* Wolbachia* in the host. Johnson hypothesized that by reducing the viral load* Wolbachia* endosymbionts enhance host survival [84]. However, since different* Wolbachia* strains affect a wide variety of insect viruses this likely suggests that the underlying mechanisms are not pathogen specific/*Wolbachia* interactions but involve putatively broad processes targeting a wide-range of viral types, including competition for resources and the upregulation of hosts' immune responses.

*(1) Wolbachia*-*Based Approach to Control Arboviruses Diseases*. A new era for controlling arboviruses started with the successful introduction of the life-shortening wMelPop-CLA* Wolbachia* strain into* Ae. aegypti* to reduce its natural populations life span [85, 86]. Primary data gathered from field trials in Australia has made it possible to validate theoretical models for* Wolbachia* population dynamics and has demonstrated the feasibility and sustainability of such a strategy to control mosquito populations and the diseases they transmit [87]. However, barriers to dispersal responsible for a slower than anticipated spread of transinfected* Aedes aegypti* mosquito in Cairns (Australia) [88] should be taken into account in future releases. Moreover, how* Wolbachia* strains of interest interfere with pathogens is a critical aspect that needs to be better understood when dealing with* Wolbachia*-based approaches. Several authors have attempted to unravel the basis of* Wolbachia* pathogen blocking. To that aim, Terradas and McGraw discussed the possible mechanistic basis of* Wolbachia*-mediated pathogen blocking and have evaluated the existence of evidences from field mosquitoes and related insects [89]. They showed that the amount of* Wolbachia* inside host cells and tissues appears to correlate with the strength of* Wolbachia*-mediated blocking. They revealed that the highly replicative* Wolbachia* strain (wMelPop) by exhibiting great cellular loads causes tissue damage thus inducing near perfect blocking in* Ae. aegypti *[89]. Another possible mode of action through which* Wolbachia* interferes with pathogen infection is by priming the host immune system, with the preactivation of the immune response which could then theoretically protect the insect from a range of pathogens. Gene regulation is another way by which* Wolbachia* modulates the host immune system as demonstrated by recent studies about the potential role of the Vago protein on the innate immune pathways of* Culex quinquefasciatus* and* Ae. aegypti *to restrict West Nile and dengue virus replication [90]. For instance, Asad et al. have shown that in* Wolbachia*-infected cells, knocking-down the Vago1 gene led to significant increases in DENV replication with no effect on* Wolbachia* density, and concluded that in* Ae. aegypti* the induction of the AeVago1 protein, mediated by* Wolbachia* in infected cells, may function as a host factor to suppress DENV replication [90].

*(2) Wolbachia*-*Based Approach to Control Malaria*. As reported in the last* World Malaria Report 2017*, despite significant progress made since 2000, the rate of decline of malaria has stalled and even reversed in some region since 2014 [91]. Reasons for this are the spread of resistance of parasite to antimalarial drugs and vectors to insecticides [4]. Beside the implementation of a strategic insecticide resistance monitoring for malaria endemic countries, the WHO's Global plan for insecticide resistance management in malaria vectors (GPIRM) highlighted also the need for the development of innovative approaches for sustainable vector control at global scale [92]. As a response to that, attention has been drawn to mosquitoes' microbiota and their potential impact on host fitness and parasite evolution [93].* Wolbachia*-mediated parasite interference in other insect systems has raised the exciting possibility of using them to control or limit the spread of malaria. However, the development of* Wolbachia*-based antimalarial strategies has been impeded by the lack of stable* Wolbachia* infections in natural anopheline populations, as well as the failure to establish stable inherited transinfections in anopheline mosquitoes. Both issues have recently been overcome with the successful establishment of a stable* Wolbachia* strain wAlbB infection in* Anopheles stephensi*, an important malarial vector in Asia [74], and the recent report of stable* Wolbachia* infections in natural populations of two important malarial vectors,* Anopheles gambiae* and* Anopheles coluzzii*, in Burkina Faso [65]. Furthermore, Shaw et al. showed that the* wAnga *strain stably infects reproductive tissues (ovaries), and certainly somatic tissues where the* Plasmodium* development occurs, and where it may effectively compete for resources or upregulate the immune response to effectively kill the malaria parasite [21]. Similar results were reported recently in Mali with a new anopheline* Wolbachia* strain (*wAnga*-Mali) [83]. Interestingly, experimental infection showed that* wAnga*-Mali has strong impact on late sporozoites stages and reduces malaria transmission [83]. Both studies showed the potential for the release of* Wolbachia*-infected mosquitoes as a promising strategy to reduce malaria transmission, but also raised the great limitation due to the apparent lack of clear Cytoplasm Incompatibility [21] to ensure released population self-sustenance in the nature. The recent discovery of native* Wolbachia* infections in 16 out of 25 wild African* Anopheles* species, including both vectors and non-vectors of malaria confirm that natural* Wolbachia* infection in anopheline mosquitoes is more common than expected [66, 67]. This offers an unprecedented opportunity to further studies the diversity of anopheline* Wolbachia* strains to identify suitable phenotypes naturally impeding the development of* Plasmodium* parasites in mosquitoes, especially among* Wolbachia *strains associated with non-malaria vectors.

## 4. Conclusions and Future Directions

This review discussed the current state of knowledge about the association between mosquitoes and* Wolbachia*, emphasizing the limitation of different mosquito control strategies and the use of mosquitoes' commensal/introduced microbiota as innovative VC intervention against mosquito-borne diseases.

In summary,

1. Several human, animal and zoonotic diseases are transmitted by mosquitoes of the* Anopheles*,* Aedes,* and* Culex* genera. Insecticide-based vector control tools/strategies are keys components in the fight against major mosquito-borne diseases.
2. The increasing emergence/ resurgence of mosquito-borne diseases such as malaria, yellow fever, dengue, chikungunya, and Zika fevers, and the spread of drug resistant parasites and insecticide resistant mosquito strains threatens the sustainability of current control methods and stresses the urgent need for the development of additional control methods for mosquito-borne diseases.
3. *Wolbachia* is one of the most promising mosquito symbionts for innovative vector control approaches.* Wolbachia* has several characteristics which can be used in such a strategy to reduce host fitness and competes or kills the pathogens.
4. *Wolbachia* was first discovered in 1924 and described in 1936 by Marshall Hertig and S. Burt Wolbach in the reproductive organs of* Culex pipiens*. The “pandemic” nature of* Wolbachia* results from their ability to manipulate host reproduction and to move horizontally across species' boundaries.
5. About 31.4% of mosquito species naturally harbour one or several* Wolbachia* strains. Moreover, it is now possible to stably transinfect mosquito vector species of medical and veterinary importance with nonnative* Wolbachia* strains which can shorten vector lifespan, limit susceptibility to infection, or induce cytoplasmic incompatibility to reduce vector density.
6. *Wolbachia*-based approach is certainly a promising innovative strategy for mosquito vector control. However, our knowledge of* Wolbachia*-mediated antiparasite mechanisms is fragmented if not entirely lacking.
7. Additional studies, including laboratory experiments, semifield, and field trial on several mosquito vector species in different geographical population urgently need to be reinforced to better understand* Wolbachia*-mediated antiparasite mechanisms and interaction between hosts and parasites but also to provide empirical data to test theoretical models for* Wolbachia* population dynamics, and demonstrate the feasibility and sustainability of* Wolbachia*-based approach approaches to control mosquito and diseases they transmit.

## Figures and Tables

**Table 1:** Taxonomic classification of Wolbachia.

**Taxa**	**Names**
Domain	*Bacteria*
Phylum	*Proteobacteria*
Class	*Alphaproteobacteria*
Subclass	*Rickettsidae*
Order	*Rickettsiales*
Family	*Rickettsiaceae*
Genus	*Wolbachia*
Type species	*Wolbachia pipientis*, Hertig 1936

**Table 2:** Taxonomic classification of mosquitoes (Diptera: Culicidae).

**Taxa**	**Names**
Kingdom	*Animalia*
Phylum	*Arthropoda*
Class	*Insecta*
Order	*Diptera*
Suborder	*Nematocera*
Infraorder	*Culicomorpha*
Superfamily	*Culicoidea*
Family	*Culicidae* Meigen, 1818
Sub-families	*Anophelinae*, *Culicinae*
Genera (112)	*Culex*, *Aedes*, *Anopheles*, etc.

**Table 3:** Main diseases transmitted by mosquito.

**Diseases**	**Pathogens**	**Genera of main vectors**	**Vertebrate hosts**	**Reservoir hosts**
**Yellow fever**	Yellow Fever virus (*Flavivirus*)	*Aedes Haemagogus Sabethes*	Humans	Monkeys
**Dengue fever**	Dengue (D1, D2, D3, D4) viruses (*Flavivirus*)	*Aedes*	Humans	Monkeys
**Chikungunya**	Chikungunya virus (*Alphavirus*)	*Aedes*	Humans	Monkeys
**Zika fever**	Zika virus (ZIKV) (*Flavivirus*)	*Aedes*	Humans	Monkeys
**Rift Valley fever**	Rift Valley fever virus (*Phlebovirus*)	*Aedes, Culex*	Sheep, Goats, Humans	Bats
**West Nile Fever**	West Nile virus (*Flavivirus*)	*Culex*	Horses, Humans	Birds
**Equine Encephalitis**	Equine Encephalitis virus (*Alphavirus*, *Flavivirus*)	*Culex*	Horses	Birds
**Japanese Encephalitis**	Japanese Encephalitis virus (*Flavivirus*)	*Culex*	Horses, Humans	Pigs and wild birds
**Saint Louis Encephalitis**	Saint Louis Encephalitis virus (*Flavivirus*)	*Culex*	Humans, Animals	Birds
**Malaria**	*P. falciparum, P. vivax, P. malariae, P. ovale, P. knowlesi*	*Anopheles*	Humans	Monkeys for* P. knowlesi*
**Lymphatic filariasis**	*Wuchereria bancrofti*, *Brugia malaya*	*Anopheles, Aedes, Culex*	Humans	Wild mammals for *B. malaya*

**Table 4:** Native Wolbachia infections in natural mosquito populations.

Host Taxa			Supergroups	Strains	GeneBank #	References
Subfamily	Genera	Species			wsp	

* Culicinae*	* Culex*	*Cx p. pipiens*	B	*wPip*	AF020060	Hertig and Wolbach 1924, Zhou *et al.*, 1998
		*Cx p. quinquefasciatus*	B	*wPip*	AF020061	Zhou *et al.*, 1998
		*Cx. brevipalpis*	A	*wBre*	AF317477	Ruang-Areerate *et al.*, 2003
		*Cx. (Eumelanomyia)* spp.	A	*wEum*	AF317480	
		*Cx. fuscocephala*	B	*wFus*	AF317481	
		*Cx. Gelidus*	B	*wGel*	AF317482	
		*Cx *(*Lophoceraomyia*) spp.	A	*WLop*	AF317490	
		*Cx. sitiens*	B	*wSit*	AF317491	
		*Cx. modestus*	B	*wPip*	-	Ricci *et al.*, 2002
		*Cq. richiardii*	B	*wCon*	-	
		*Cx. torrentium*	B, C	*wPip, wDi*	-	
	*Aedes*	*Ae. albopictus*	A, B	*wAlbA, wAlbB*	AF020059, AF020059	Wright and Wang 1980; Zhou *et al.*, 1998
		*Ae. albotaeniatus*		*wAlbo*	AF317475	Ruang-Areerate *et al.*, 2003
		*Ae*. *craggi*		*WCrag*	AF317478	
		*Ae. novoniveus*		*wNov*	AF317484	
		*Ae. niveus*		*wNiv*	AF317485	
		*Ae. pseudalbopictus, *		*wPseu*	AF317487	
		*Ae. perplexus*		*wPerp*	AF317486	
		*Ae*. *cooki*			-	Yen 1975; Dean & Dobson (2004); Takken & Koenraadt 2013
		*Ae*. *polynesiensis*			-	
		*Ae*. *riversi*			-	
		*Ae. cinereus*	C	*wDi*	-	Ricci *et al.*, 2002
		*Ae. detritus*	C	*wDi*	-	
		*Ae. geniculatus*	C	*wDi*	-	
		*Ae. punctor*	B, C	* wPip, wDi*	-	
		*Ae. fluviatilis*	B	*wFlu*	GQ981315	Moreira et al., 2009
						
	*Armigeres*	*Arm. subalbatus*	A	*wSub*	AF317488	Ruang-Areerate *et al.*, 2003
					
	*Mansonia *	*Mn. uniformis*	B	*wUnif*	AF317493	
		*Mn. indiana*	B	*wInd*	AF317492	
					
	*Coquillettidia*	*Cq. crassipes*	B	*wCra*	AF317479	
						
*Anophelinae*	*Anopheles*	*An. gambiae*	A, B	*wAnga-BF, wAnga-Mali*	KJ728739-MF944223	Baldini *et al.*, 2014; Shaw *et al.*, 2016; Gomes *et al.*, 2017
		*An. coluzzii*	A, B	*wAnga-BF, wAnga-Mali*	KJ728755-MF944223	
		*An. arabiensis*	A, B	*wAnga*	KJ728739- KJ728755	Shaw *et al.*, 2016
		*An. funestus*	A, B	*wAnfu*	-	Niang *et al.*, 2018 [68], Ayala *et al.*, 2018
		*An. carnevalei*	A,B	*-*	*-*	Ayala et al., 2018
		*An. coustani*	B	*-*	*-*	
		* An. hancocki*	B	*-*	*-*	
		*An. implexus*	B	*-*	*-*	
		* An. jebudensis*	B	*-*	*-*	
		*An. marshallii *	B	*-*	*-*	
		*An. moucheti *	B	*-*	*-*	
		*An. nigeriensis*	B	*-*	*-*	
		*An. nili*	B	*-*	*-*	
		*An. paludis*	B	*-*	*-*	
		*An. vinckei*	A,B	*-*	*-*	
